# Clinical Assessment of Eye Movement Desensitization and Reprocessing in Memory Distress: Protocol for a Double-Blinded Randomized Controlled Trial

**DOI:** 10.2196/38552

**Published:** 2023-05-12

**Authors:** Nazanin Babaei, Camrie Kerry, Kisha Goode, Kevin Dang, Parsa Mirzadeh, Meysam Pirbaglou, Megan A Kirk, Paul Ritvo

**Affiliations:** 1 School of Kinesiology and Health Science York University Toronto, ON Canada; 2 Yale Center for Emotional Intelligence Yale School of Medicine New Haven, CT United States

**Keywords:** EMDR variants, eye movement desensitization reprocessing, flash technique-EMDR, posttraumatic stress disorder

## Abstract

**Background:**

Exposures to “traumatic” events are widespread and can cause posttraumatic stress disorder (PTSD). Cognitive behavioral therapy and eye movement desensitization and reprocessing (EMDR) are frequently used and validated behavioral PTSD treatments. Despite demonstrated effectiveness, highly upsetting memory reactions can be evoked, resulting in extensive distress and, sometimes, treatment dropout. In recent years, multiple treatment approaches have aimed at reducing such upsetting memory reactions to traumatic memories while therapeutic progress proceeds. One of these methods, the flash technique (FT), a modification of standard EMDR (S-EMDR), appears effective in distressing memory reduction. This study will examine FT-EMDR and S-EMDR efficacies when both methods are delivered via web-based video.

**Objective:**

This study aims to assess the relative efficacy of (web-based) FT-EMDR versus S-EMDR in reducing the PTSD symptoms, anxieties, and depression associated with traumatic memories at postintervention and 1-month follow-up.

**Methods:**

This double-blinded, web-based, 2-arm randomized controlled trial will employ self-report outcomes. A total of 90 participants will be identified from the web-based CloudResearch platform and randomly allocated to the experimental or comparison group. Inclusion criteria are as follows: (1) approved for engagement by the CloudResearch platform; (2) 25-60 years of age; (3) residing in Canada or the United States; (4) a recalled disturbing memory of an event >2 years ago that has not repeated and was moderately or more upsetting during occurrence; (5) memory moderately or more upsetting at baseline and not linked to an earlier memory that is equally or more than equally disturbing. Exclusion criteria are bipolar disorder, borderline personality disorder, obsessive-compulsive disorder, schizophrenia, substance abuse or addiction in the past 3 months, suicidal ideation, and suicide attempt in the past 6 months. Interventions include guided video instruction of full FT or guided video of EMDR. Outcome measures are as follows: Primary outcome is PTSD symptoms that are measured by the PTSD Checklist for DSM-5 (Diagnostic and Statistical Manual of Mental Disorders-5) at 1-month follow-up. Secondary outcomes are State Anxiety subscale of State-Trait Anxiety Inventory at baseline, postintervention, and 1-month follow-up; Trait Anxiety subscale of State-Trait Anxiety Inventory; depression (Patient Health Questionnaire-9); and Positive and Negative Affect Schedule measured at 1-month follow-up.

**Results:**

If, at 1-month follow-up, the web-based FT-EMDR intervention is more effective in reducing PTSD symptoms (as measured by the PTSD Checklist for DSM-5) than EMDR, it may help reduce traumatic memory distress in multiple contexts.

**Conclusions:**

This randomized controlled trial will advance current understandings of PTSD symptoms and interventions that target traumatic memory–related distress.

**Trial Registration:**

ClinicalTrials.gov NCT05262127; https://clinicaltrials.gov/ct2/show/NCT05262127

## Introduction

Posttraumatic stress disorder (PTSD) can occur after direct or indirect exposure to traumatic events with frequent symptoms of reexperiencing, avoidance, cognitive-mood alterations, and heightened arousal or reactivity [1]. Traumatic exposures are widespread [2-4], and a study of 2991 Canadian adults indicated that 76% of participants reported one or more traumatic exposures of the type that can lead to diagnosed PTSD [5]. The prevalence of diagnosed PTSD is ~3.9% worldwide and 10% in Canada (~3.7 million people) [5-7]. Disturbing memory retention can lead to diagnosable PTSD, while adversely impacting psychological and physiological well-being [8-10]. Innovative interventions that reduce memory-based disturbance can prevent PTSD diagnoses and improve current status.

Pharmacological PTSD treatments include antidepressants, sympatholytic drugs, antipsychotics, anticonvulsants, and benzodiazepines [11-13]. Despite the availability of these agents, their limited efficacy has motivated adjunctive behavioral interventions [14,15]. A meta-analysis of PTSD treatment indicated that psychological therapies were generally more effective than drug therapies in symptom reduction [14].

Several of the most prominent methods addressing “traumatic” memories are trauma-focused cognitive behavioral therapy, cognitive processing therapy, prolonged exposure, and eye movement desensitization and reprocessing (EMDR) [16-18]. Other exposure-based treatments designed for PTSD are narrative exposure therapy and written exposure therapy [19]. In traditional EMDR, patients follow the horizontal movement of the therapist’s index finger while recalling details of the traumatic memory [20]. The attention devoted to finger movement during recall has been proposed to reduce the intensity and intrusiveness of memory disturbance [20-24]. EMDR is now widely supported as a treatment for PTSD and is being tested for other mental health conditions (eg, generalized anxiety disorders, addiction, and depression) [25,26].

The mechanisms by which EMDR delivers benefits are controversial [27-29] and differ substantially from those proposed for other psychological therapies [30-32]. The divergence maps onto a frequent limitation acknowledged in exposure approaches, namely, traumatic memory confrontation, can generate high arousal levels, emotional distress, dissociation, and treatment dropout [33,34]. The novel variant of EMDR for PTSD investigated in the proposed study, the flash technique-EMDR (FT-EMDR) [35,36], emphasizes, in combination with standard EMDR (S-EMDR) procedures, the use of a positive engaging focus (PEF), which is recalled and imagined while a right-hand-left-hand alternate knee tapping proceeds [35-38]. Although several explanations for the mechanisms mobilized by this method are proposed, their identification is secondary to our goal of assessing efficacy within a double-blinded randomized controlled trial (RCT) that compares S-EMDR with FT-EMDR. If the FT variant demonstrates greater efficacy, mechanism identification and measurement may proceed more clearly.

As FT is a novel EMDR variant, the related research is in its early stages. Nonetheless, our study will follow prior findings that suggest FT efficacy in reducing the distress associated with disturbing memories [35-38]. If efficacy is indicated, one or both approaches could be used in geographic areas that require more immediate treatment needs, such as when warfare or natural disasters evoke traumatic reactions that are more prevalent.

This study has following three objectives: (1) to evaluate whether web-based delivery of FT is more effective in reducing PTSD symptoms than web-based S-EMDR at 1-month follow-up measured by the PTSD Checklist for DSM-5 (Diagnostic and Statistical Manual of Mental Disorders-5) (PCL-5); (2) to assess whether web-based delivery of FT-EMDR is more effective in reducing state anxiety symptoms than S-EMDR at postintervention and 1-month follow-up; and (3) to evaluate whether web-based delivery of FT-EMDR is more effective in reducing depressive symptoms than S-EMDR at postintervention and 1-month follow-up.

## Methods

### Overview

Previous studies of FT-EMDR have indicated that guided in-person FT effectively reduces subjective units of distress caused by distressing memories. These studies were limited due to a lack of randomization and adequate control comparison. This study will be an assessor-blinded, 2-arm (experimental and control) randomized controlled trial, which minimizes placebo effects and implements an adequate sample size and psychometric measurement. This study is expected to have an overall duration of 3 months. The length of study for each participant will be 4 weeks. The recruitment period is estimated to last for 2 months.

### Ethical Considerations

The study was approved by the Research and Ethics Board at York University (087/2020) and is registered with ClinicalTrials.gov (NCT05262127).

### Informed Consent

After eligibility is confirmed by the investigator, web-based informed consent will be obtained from participants. Each participant will review approved informed consent documents where guaranteed privacy and confidentiality protections are specified, along with assurances that all study data will be anonymized and deidentified. Each participant is compensated US $17 for participation. Participants will be informed about the scientific benefits of the study; however, they are informed that their participation is voluntary, and they have the right to terminate their participation at any time. The signed form will be retained as part of the study records.

### Sample Size

#### Overview

With the assistance of the Institute for Social Research at York University, the sample size was calculated for a 3-way repeated measures ANOVA. Sample size calculation was conducted using G*Power 3.1.9.4. The options in G*Power 3.1.9.2 were as follows: (1) test family: “*F* test”; (2) statistical test: “ANOVA: Repeated measures, between factor interaction”; and (3) type of power analysis: “A priori: Compute required sample size–given α, power, and effect size.” An a priori power analysis was completed using a moderate effect size (*F*=0.25), 0.8 power (1β error probability), and an α error probability at *P*<.05. G*Power 3.1.9.4 determined that a minimum sample size of 37 participants per group provides ample testing power. Considering a 20% (n=7.4) oversampling to account for potential participant dropout and experimental error, a proposed minimum sample size of 44 per group is deemed adequate to detect within-group and between-group differences based on a repeated measures design.

#### Inclusion Criteria

Participants must pass the CloudResearch platform’s standardized assessment of *attention*, *engagement*, and *language comprehension* for study participation [39]. Language comprehension and engagement are assessed by identifying synonyms, reading articles, and answering comprehension questions. Additional attentional competencies are assessed by evaluating factual correctness in answers to demographic questions (eg, “I work 28 hours in a typical workday”). In addition, the CloudResearch platform screening is based on a recorded history of providing high-quality data in prior study participation.

In the investigator-based criteria, participants must (1) be between 25 and 60 years of age and maintain residence in the United States or Canada; (2) be able to identify a memory regarding an event that occurred more than 2 years ago and has not been since repeated; (3) identify the memory as moderately upsetting, or more than moderately upsetting, when it occurred; (4) clearly recall the identified memory; (5) find the memory to be still moderately upsetting, or more than moderately upsetting, when recalled; and (6) identify the memory as not tied to an earlier memory that is equally or more disturbing.

Since participants are exposed to only 1 brief session, processing a series of upsetting memories is unreasonable and potentially harmful. Hence, the inclusion of only a single distressing memory, which should be distant from the participation date, was carefully implemented to minimize emotional arousal.

#### Exclusion Criteria

Exclusion criteria are as follows: (1) individuals who disclose a past or present self-reported diagnosis of bipolar disorder, borderline personality disorder, obsessive-compulsive disorder, schizophrenia, or a substance abuse or addiction in the past 3 months; and (2) individuals who disclose having suicidal ideation or who have attempted suicide in the past 6 months prior to the study.

### Recruitment Plan

Participants will be recruited from a web-based platform (CloudResearch) [40]. Potentially eligible participants will be prescreened and eligibility assessed. After eligibility screening, they will be invited to a Zoom meeting where the study investigator further explains the study procedures. If the participant is interested, written consent will be obtained prior to randomization. The investigator will perform electronic randomization, with study IDs blindly assigned to experimental and control group participants. Study ID information will be recorded on an excel sheet as well as on the SurveyMonkey platform. After a participant completes baseline questionnaires, blinded research assistants will play a taped version of the video assigned to the participant in accordance with the randomization plan.

The randomly assigned participants will proceed to watch a 15-minute guided video of either FT-EMDR (experimental condition) or S-EMDR (comparison condition) in a quiet environment. Assessments will take place at pre- and postintervention and 1-month follow-up.

A streaming recruitment method will be employed where the study description is made available on the internet (CloudResearch platform) for a duration of 15 minutes prior to a participation-designated interview time slot. This will enable potential participants to enter the study description link, read a description of the study format, and pass the eligibility criteria before proceeding further if interested in study participation.

The additional steps to “streaming recruitment” require specifying how many participants can access the experiment’s Zoom link at a given time, up to a maximum of 15. When more than one subject uses the link to enter the Zoom waiting room, the researcher will allow the first participant to enter the Zoom study meeting, while the remaining waiting room occupants are thanked for their interest and prompted to sign off. Once 1 eligible participant enters the video interview, the Zoom meeting will be locked, and no additional participants can access or join.

### Procedure

This study will require 2 contact commitments from participants during a 4-week period. The first interaction will include gaining consent, completing preintervention psychometric questionnaires, and then watching and engaging in the 15-minute either intervention or control video. Subsequently, participants will complete postintervention measures and are provided with a debriefing statement.

The second interaction will be a follow-up of intervention outcomes 1 month after the initial interaction. A SurveyMonkey link will be sent out to participants via the CloudResearch platform, which will include all primary and secondary outcome measures. Both participants and research assistants will be blinded to experimental and control conditions throughout the trial.

### Interventions

#### Experimental Group

After a participant consents and completes demographic and baseline psychometric questionnaires, a “blinded” research assistant will share the computer screen and play a taped video of FT-EMDR containing vocalized and viewable instructions by licensed EMDR practitioners. Participants will be asked to follow the instructions with their full attention in a distraction-free environment. Participants assigned to the experimental condition will be invited by the video instructor to perform the following additional tasks: (1) identify a traumatic memory and rate its disturbance on a numerical scale that extends from 0 (no disturbance at all) to 10 (most disturbing)—This is often referred to as the Subjective Units of Distress Scale (SUDS) [41]; (2) identify a PEF, which can include a person, vacation, pet, favorite activity, or favorite music that is immediately gratifying when recalled or is a reminder of a past gratifying experience; (3) rhythmically tap knees while thinking of the selected PEF; periodically, the video instructor will say the word “flash,” prompting 4-6 rapid blinks of the eyes, simultaneously establishing the blink rhythm and duration; (4) continuously envision the proposed PEF after blinking; and (5) respond to periodic check-ins concerning changes in the memory experience.

At the end of the video, the instructor will ask participants to rate their target memory once again on the SUDS. The assigned research assistant will collect SUDS reports at baseline and postintervention.

The PEF is considered essential to the FT-EMDR approach component. Participants will be instructed to immerse themselves into their PEF while rhythmically tapping their knees; however, they will not be instructed to avoid and suppress their target memory. Conversely, participants will be encouraged to bring their attention back to their PEF once distracted by the target memory.

#### Comparison Group

Members of the comparison group will be exposed to a pretaped video following similar instructions by the same licensed EMDR practitioners as the experimental group. In a procedure that is identical to the experimental group, participants will be instructed to identify a traumatic event as a target memory. The concept of PEF, however, will not be mentioned, precluding any engagement with or use of it. Instructions will specify that participants should only focus on their traumatic memory and recall as many details as possible. The duration and rhythm of tapping and blinking are identical to what is done with the experimental group, and SUDS measurements are taken in a similar manner. The comparison group protocol will closely follow the S-EMDR format, where clients generate back-and-forth eye movements while focusing on vivid recalls of the traumatic event.

### Outcome Measures

#### Primary Outcome

The primary outcome measure will be the PCL-5 [42]. It is a 20-item self-report instrument measuring the presence and severity of PTSD symptoms. Each item is rated on a 5-point Likert scale ranging from 0 (not at all) to 4 (extremely), indicating how much each symptom has bothered the respondent in the past month. Higher scores indicate more severe PTSD-like symptoms, and a score higher than 31-33 alerts to a possible PTSD diagnosis.

#### Secondary Outcomes

Four self-reported secondary outcome measures will be employed only at baseline and 1-month follow-up. All outcome measures will be carried out on the internet via SurveyMonkey links.

The State-Trait Anxiety Inventory (STAI) Form Y-1 (S-STAI, where S is state subscale) [43] is a 20-item self-report scale that measures subjective tensions, apprehension, nervousness, worries, and physiological arousal. The customary cutoff score for probable clinical levels of anxiety is 40 and above. Trait anxiety is measured by STAI Form Y-2 (T-STAI; trait subscale) [43], which is a 20-item self-report scale. The trait scale measures how people generally feel and it is the most widely used measures for assessing anxiety in clinical and experimental settings [44].

Patient Health Questionnaire-9 (PHQ-9) [45] is used to assess depression levels. This questionnaire has 9 items rated on a 5-point Likert scale from 0 (not at all) to 4 (nearly every day). Similarly, expected ranges for nonminimal, mild, moderate, moderately severe, and severe depression are 0-4, 5-9, 10-14, 15-19, and 20-27.

**Figure 1:**
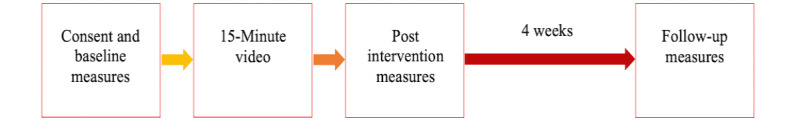
Visual representation of study procedure. Baseline measures: State Subscale-State-Trait Anxiety Inventory (S-STAI), Trait Subscale-STAI (T-STAI), Subjective Units of Distress Scale (SUDS), Posttraumatic Stress Disorder Checklist for DSM-5 (PCL-5), Patient Health Questionnaire-9 (PHQ-9), and Positive and Negative Affect Schedule (PANAS). Postintervention measures: S-STAI and SUDS. Follow-up outcome measures: S-STAI, T-STAI, PCL-5, PHQ-9, and PANAS.

Positive and Negative Affect Schedule (PANAS) [46] is a 20-item questionnaire that measures positive (10 items) and negative (10 items) affects. Each item is scored with a 5-point Likert scale ranging from 1 (not at all) to 5 (very much). Higher scores on a positive scale translate to higher levels of positive affect, whereas lower scores on negative affect suggest low levels of negative affect. This reliable and valid self-report scale is brief, easily administered, highly internally consistent, and stable at appropriate levels over about as long as a 2-month time period [46]. Visual representation of the study procedures is shown in Figure 1.

### Hypotheses

FT-EMDR (the experimental condition) will be significantly more effective than S-EMDR in reducing self-report scores on the PCL-5 at 1-month follow-up to the intervention than traditional EMDR.

FT-EMDR (the experimental condition) will be significantly more effective than S-EMDR in reducing self-report scores on the S-STAI at postintervention and 4-week follow-up than traditional EMDR.

FT-EMDR will be significantly more effective than S-EMDR in decreasing self-report scores on the PHQ-9 at 1-month follow-up.

### Statistical Analyses

Statistical analysis will include the calculation of descriptive statistics for demographic and psychological characteristics at baseline, postintervention, and the follow-up period. Study outcomes will be presented as means and SDs for numeric variables and frequencies and percentages for categorical variables. In addition, potential between-group differences at baseline will be evaluated using *t* tests for numeric variables and chi-square tests of independence for categorical variables. Significance will be set at *P*≤.05 and will be presented with *r* values for each questionnaire.

To evaluate the effects of the intervention on primary study outcome (PCL-5) and secondary outcomes (PHQ-9, S-STAI, Trait Subscale-State-Trait Anxiety Inventory, PANAS-Positive, and PANAS-Negative), linear mixed models (LMM) will be employed with an AR (1) covariance structure and random intercepts for each study participant to allow for both fixed and random effects. The LMM assumes that the outcome measure is normally distributed. All models will include adjustments for age and sex (among other psychological characteristics). Statistically significant group×time interactions, indicating between-group changes in outcomes, will be followed by simple main effect evaluation. Cohen d effect sizes for between-group and within-group comparisons will also be calculated according to procedures outlined in Lakens [47]. All analyses will be conducted using RStudio [48] and associated statistical packages, including nlme [49], psych [50], and ggplot2 [51].

### Missing Data

Missing data in the context of this study occurs if study participants miss an assessment at the immediate postintervention or drop out prematurely before the 1-month follow-up assessment. Missing observations, in addition to specific reasons for dropout, will be monitored and presented as part of the associated study flow diagram. The LMM does not require imputations and uses a restricted maximum likelihood strategy to estimate study parameters. Restricted maximum likelihood produces unbiased estimates of variance and covariance variables.

## Results

Our findings are scheduled for a full analysis, with expectations that analyses will be completed by July, 2023. Our intention is to publish results in peer-reviewed journals.

## Discussion

In this study, 2 videotaped EMDR variants are compared for efficacy within a double-blinded RCT. They are conveyed by registered EMDR therapists in a web-based format and compared for efficacy using standard psychometric scales at postintervention and 1-month follow-up. The innovations addressed are (1) use of videotaped approaches conveyed through web-based contacts; and (2) different EMDR approaches, varying by insertion of a PEF (in FT-EMDR) and its absence (in S-EMDR). Aside from the inclusion or omission of the PEF, the approaches to EMDR employed in this study are nearly identical. It is important to note that a qualified clinical psychologist will be available while participants undergo either one of these interventions in case of psychological disturbance.

If study findings reveal the efficacy of these interventions, one or both innovations could be potentially useful when individuals with disturbing memories are distant geographically from areas where face-to-face encounters with EMDR therapists can be arranged. Some individuals may also find the typical professional fees too expensive. The web-based alternative is likely to be considerably less expensive. Given the divergences from routine EMDR services, these interventions might be considered stop-gap measures. Nonetheless, this trial provides an initial evaluation of these alternative (web-based) treatments.

Other investigators interested in PTSD treatment have been experimenting with web-based and video-based tools [52,53]. For example, an actuator- and web-based system based on EMDR principles was tested to reduce anxiety, distress, and negative cognitions [52]. This autonomous system used video, tactile, and audio *actuators* and an artificial intelligence “chatbot.” In a pilot single-arm study of 31 subjects selected for moderate baseline scores on the Impact of Events Scale-Revised [54] and the STAI [43], subjects demonstrated significant reductions on the Impact of Events Scale-Revised and STAI following a single session of intervention. The system directed by the investigators recruited 24 participants and exposed them to a 1-session intervention, using a “Blind 2 Therapist” EMDR protocol as a videoconference psychotherapy format that included nondisclosure of the traumatic target memory [53]. This group also reported significant reductions on several self-report scales, notably a SUDS and Validity of Cognition Scale [21], designed to evaluate cognitive structure on emotional and somatic levels. Significant reductions in several self-report instruments were found at postintervention, 1-month, and 6-month follow-up.

The limitations of both studies are the nonblinded, posttreatment assessments that are invariably susceptible to placebo responding. In our study, the double-blinded RCT design aims to limit the placebo response. The data we derive must be regarded as preliminary and preparatory to follow-up studies that, optimally, would include several additional components: (1) a larger group of EMDR-trained therapists to convey interventions (so as to compare results across varying backgrounds, training, and gender); (2) comparisons between participants recruited using the internet (in a similar manner) versus participants recruited through clinics (to compare results regarding initial contact mode). Other acknowledged study limitations include the sole employment of self-report measures and the use of only postintervention and 1-month follow-up, rather than a more extensive series of longitudinal measures.

In conclusion, the study represents a step forward in evaluating web-based, video-taped interventions, which may prove useful where large populations are traumatically exposed (ie, through environmental disasters and warfare) and routine psychological therapy is difficult to deliver. Once widely available, a combination of EMDR variants with other technological tools could possibly broaden the opportunity for more accessible therapy.

## Abbreviations

DSM-5: Diagnostic and Statistical Manual of Mental Disorders-5
EMDR: eye movement desensitization and reprocessing
FT: flash technique
FT-EMDR: flash technique-eye movement desensitization and reprocessing
LMM: linear mixed models
PANAS: Positive and Negative Affect Schedule
PCL-5: PTSD Checklist for DSM-5
PEF: positive engaging focus
PHQ-9: Patient Health Questionnaire-9
PTSD: posttraumatic stress disorder
RCT: randomized controlled trial
S-EMDR: standard eye movement desensitization and reprocessing
S-STAI: State Subscale-State-Trait Anxiety Inventory
STAI: State-Trait Anxiety Inventory
SUDS: Subjective Units of Distress Scale

## Data Availability

Following analysis, an anonymized data set will be made available to interested fellow investigators by the corresponding author upon reasonable request.
